# Is Reducing the Obesity Epidemic in Children and Adolescents Really a Mission Impossible?

**DOI:** 10.3390/nu17030592

**Published:** 2025-02-06

**Authors:** Simonetta Genovesi, Antonina Orlando, Marco Giussani

**Affiliations:** 1 School of Medicine and Surgery, Milano-Bicocca University, 20126 Milan, Italy; 2 Istituto Auxologico Italiano, Istituto Ricovero Cura Carattere Scientifico (IRCCS), 20135 Milan, Italy; a.orlando@auxologico.it (A.O.); m.giussani@auxologico.it (M.G.)

The prevalence of pediatric excess weight has reached such levels that there are fears of a sharp increase in associated noncommunicable diseases when today’s children become adults. Most children with obesity are likely to become obese adults [1], so the prevalence of obesity in the future may also increase. In addition, children who are in excess weight today, if left untreated, will long be exposed to a risk factor for their future well-being. Diseases associated with obesity involve almost every organ system. If we narrow the field to cardiovascular diseases alone, much evidence shows that hypertension, dyslipidemia, hyperuricemia, and insulin resistance have a much higher prevalence in excess-weight children and adolescents than in their normal-weight peers [2].

Obesity is therefore a disease that can affect the health of children and adolescents and their hope for a healthy life when they will become adults [3]. Physicians, and pediatricians in particular, are aware of this problem. In contrast, many parents of children with obesity think that excess weight, even severe excess weight, is a transient condition that will resolve as they grow. To make a diagnosis of obesity is not difficult: all that is needed is a scale, a statimeter, and the ability to calculate the body mass index (BMI) and to interpret it correctly through validated growth curves [4,5]. In addition, measuring waist circumference indexed for height can easily give important information about the amount of visceral fat [6]. These simple tools allow proper classification of the child’s weight status. Once the diagnosis is made, however, many complex factors come into play: the role of genetics and epigenetics, that of environmental factors, that of dietary habits, environmental pollution, low physical activity, and a sedentary lifestyle. A point of paramount importance is what preventive actions should be put into action, both at the social and political level and at the clinical level.

Diet quality plays a key role in both the development and treatment of pediatric obesity. Despite evidence of the beneficial effects of some healthy diets such as the Mediterranean diet [7], the dietary pattern that unfortunately seems to be gaining popularity worldwide is that of the Western diet, which is characterized by a major use of junk food and ultra-processed foods. In recent decades, there has been a profound change in traditional eating habits through the widespread use, even in children, of foods treated with physical and chemical processes to change their taste and consistency with the aim of increasing their sales and consumption. This change, on the one hand, has favored the development of obesity, while on the other hand it raises concerns about the long-term health effects of such deeply modified foods [8,9]. One study, included in this Special Issue, shows that the nutritional skills of parents do not seem to be sufficient to lead to the improved body weight of either the parents themselves or their children. The study shows that normal-weight parents, better economic conditions, and the higher cultural level of the family are instead more important factors in determining children’s weight than nutritional knowledge [10]. This study requires reflection on the fact that providing nutrition information to parents may not be sufficient, without an overall improvement in the cultural and socioeconomic conditions of families, to affect the problem of excess obesity in children.

Another important point, often overlooked, is the environmental and behavioral factors associated with obesity. Children of severe excess weight have poor exercise tolerance [11]. In addition, they are often precluded from playing sports or their participation is made difficult by their own peers who bully them [12]. This phenomenon leads to the exclusion of children and adolescents with obesity from group activities and sports practice, which has a negative effect on body weight and self-estimation. All this results in children and adolescents with obesity often being particularly sedentary and devoted to video games. Both of these can negatively affect another important factor, the quantity and quality of sleep. [13,14]. The relationship between obesity, sleep, and nutrition is complex, and these factors are mutually interdependent. Poor sleep quality is among the factors that may promote the development of obesity, but the opposite may also be true. In fact, children with obesity more frequently manifest sleep disorders than children of normal weight. All alterations in different aspects of sleep, such as quantity, quality, efficiency, and timing, can have a bearing on the development of obesity [15]. All these factors should therefore be carefully looked for in the history of an excess-weight child and, if present, corrected. Often, poor sleep duration, constant nighttime awakenings, or time spent in front of a video game instead of sleeping are associated with excessive consumption of ultra-processed or junk foods. These foods are rich in free sugars, saturated or hydrogenated fatty acids, stimulants (caffeine, chocolate, tea) that impair falling asleep and achieving regular sleep. On the other hand, a proper diet, rich in fruits and vegetables in accordance with the principles of the Mediterranean diet; the regularity of mealtimes, sleep, and wakefulness; and the performance of regular physical activity have positive effects on all components of sleeping in children.

From the above, it is clear the complexity of early and effective intervention aimed at preventing the onset of obesity in children of normal weight and reducing excess weight in children in whom the problem is already present. As mentioned above, in addition to being a health risk factor in itself, obesity is also a condition associated with several cardiovascular risk factors, disease conditions that are much more prevalent in excess-weight children than in their normal-weight peers. Included in the Special Issue is a review that, in addition to presenting an update of the literature related to the dietary behavioral treatment of pediatric excess weight, addresses in detail the measures to be taken according to the cardiovascular risk profile associated with a child’s obesity, to be added to those already planned to counteract obesity [16]. In this way, dietary intervention can be tailored to the individual child’s risk profile. Recent data show that this approach is effective at reducing excess weight [17] and the prevalence of metabolic alterations [18] and early cardiac damage [19]. Treating pediatric obesity is a difficult challenge. It seems that the most effective models of intervention are those involving multiple specialists in a multidisciplinary team [20]. However, it seems difficult to imagine that such an approach could be extended to all children who need it, given the very high prevalence of obesity in children. It is therefore important that obesity prevention begin as early as possible, with the careful monitoring of children’s growth in order to implement early interventions if necessary. In the presence of very severe excess weight, whether associated or not with other cardiovascular risk factors, the pediatrician alone may not be fully equipped to deal with the management of the young patient. In these cases, an interdisciplinary intervention is recommended, involving, in addition to the central figure of the pediatrician, the participation of nutritionists, cardiologists, experts in metabolic pathologies, sports physicians, and specialists capable of dealing with the many psychological aspects possibly related to the problem.

This Special Issue cannot, of course, cover all the complexity of the problem of how to treat pediatric obesity, but it strives to make a contribution relevant to physicians who are faced with the problem, providing a number of insights and also several suggestions that may be of use in clinical practice.
